# Supplementary material for: Prevalence of Elevated Blood Triglycerides and Associated Risk Factors: Findings from a Nationwide Health Screening in Mongolia
Source: Int J Environ Res Public Health. 2024 Nov 25;21(12):1559. doi: 10.3390/ijerph21121559 (PMC11675759; doi:10.3390/ijerph21121559)
Supplement: Supplementary file 1 [file ijerph-21-01559-s001.zip › ijerph-3212457-supplementary.pdf]

Table S1. Characteristics of study population.

Findings	Triglyceride Category and Tukey's Five-Number Summary (Minimum, First Quartile, Median, Third Quartile, Maximum)		
	**Normal**	**Borderline High**	**High**
Age (years)	[20, 30, 40, 50, 80]	[22, 35, 45, 55, 82]	[23, 37, 48, 57, 84]
BMI (kg/m²)	[18.0, 23.5, 26.0, 28.5, 35.0]	[20.0, 26.5, 28.5, 31.0, 36.5]	[21.0, 27.0, 29.1, 32.5, 37.0]
Waist Circumference (male, cm)	[65, 80, 86.5, 93, 110]	[70, 85, 93.5, 100, 115]	[72, 88, 95.5, 102, 118]
Waist Circumference (female, cm)	[60, 75, 83.5, 91, 105]	[65, 80, 90.6, 97, 110]	[67, 83, 91.2, 99, 112]
Systolic BP (mmHg)	[90, 110, 118.9, 130, 150]	[100, 115, 124.3, 135, 160]	[105, 120, 125.0, 140, 165]
Diastolic BP (mmHg)	[60, 70, 76.7, 85, 100]	[65, 75, 80.1, 90, 105]	[67, 78, 81.0, 92, 107]
Total Cholesterol (mmol/L)	[3.5, 4.5, 4.95, 5.5, 6.8]	[4.2, 5.1, 5.54, 6.0, 7.2]	[4.3, 5.2, 5.51, 6.1, 7.5]

Data are presented as minimum, first quartile, median, third quartile and maximum.

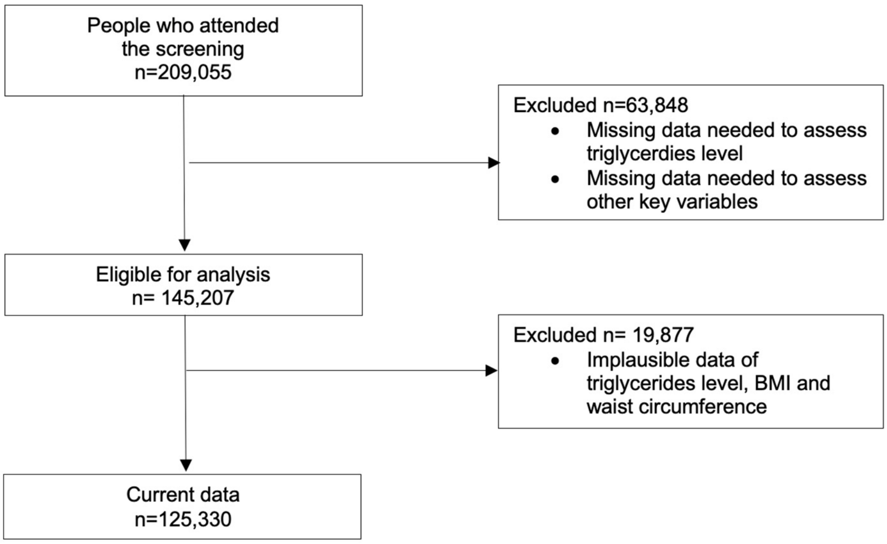

Figure S1. Flowchart of the study population.
